# Determinants of the maximal functional reserve during repeated supramaximal exercise by humans: The roles of Nrf2/Keap1, antioxidant proteins, muscle phenotype and oxygenation

**DOI:** 10.1016/j.redox.2023.102859

**Published:** 2023-08-22

**Authors:** Victor Galvan-Alvarez, Marcos Martin-Rincon, Angel Gallego-Selles, Miriam Martínez Canton, NaDer HamedChaman, Miriam Gelabert-Rebato, Mario Perez-Valera, Eduardo García-Gonzalez, Alfredo Santana, Hans-Christer Holmberg, Robert Boushel, Jostein Hallén, Jose A.L. Calbet

**Affiliations:** a Department of Physical Education, University of Las Palmas de Gran Canaria, Campus Universitario de Tafira s/n, Las Palmas de Gran Canaria, 35017, Spain; b Research Institute of Biomedical and Health Sciences (IUIBS), University of Las Palmas de Gran Canaria, Paseo Blas Cabrera Felipe "Físico" s/n, 35017, Las Palmas de Gran Canaria, Spain; c Department of Exercise Physiology, Faculty of Sports Sciences, University of Mazandaran, Babolsar, Mazandaran, Iran; d Complejo Hospitalario Universitario Insular-Materno Infantil de Las Palmas de Gran Canaria, Clinical Genetics Unit, 35016, Las Palmas de Gran Canaria, Spain; e Department of Health Sciences, Luleå University of Technology, Sweden; f School of Kinesiology, Faculty of Education, The University of British Columbia, Vancouver, BC, Canada; g Department of Physical Performance, The Norwegian School of Sport Sciences, Postboks, 4014 Ulleval Stadion, 0806, Oslo, Norway

**Keywords:** Fatigue, ROS, Free radicals, Keap1, Nrf2, Performance, Ischaemia, High-intensity exercise

## Abstract

When high-intensity exercise is performed until exhaustion a “functional reserve” (FR) or capacity to produce power at the same level or higher than reached at exhaustion exists at task failure, which could be related to reactive oxygen and nitrogen species (RONS)-sensing and counteracting mechanisms. Nonetheless, the magnitude of this FR remains unknown. Repeated bouts of supramaximal exercise at 120% of VO_2_max interspaced with 20s recovery periods with full ischaemia were used to determine the maximal FR. Then, we determined which muscle phenotypic features could account for the variability in functional reserve in humans. Exercise performance, cardiorespiratory variables, oxygen deficit, and brain and muscle oxygenation (near-infrared spectroscopy) were measured, and resting muscle biopsies were obtained from 43 young healthy adults (30 males). Males and females had similar aerobic (VO_2_max per kg of lower extremities lean mass (LLM): 166.7 ± 17.1 and 166.1 ± 15.6 ml kg LLM^−1^.min^−1^, P = 0.84) and anaerobic fitness (similar performance in the Wingate test and maximal accumulated oxygen deficit when normalized to LLM). The maximal FR was similar in males and females when normalized to LLM (1.84 ± 0.50 and 2.05 ± 0.59 kJ kg LLM^−1^, in males and females, respectively, P = 0.218). This FR depends on an obligatory component relying on a reserve in glycolytic capacity and a putative component generated by oxidative phosphorylation. The aerobic component depends on brain oxygenation and phenotypic features of the skeletal muscles implicated in calcium handling (SERCA1 and 2 protein expression), oxygen transport and diffusion (myoglobin) and redox regulation (Keap1). The glycolytic component can be predicted by the protein expression levels of pSer^40^-Nrf2, the maximal accumulated oxygen deficit and the protein expression levels of SOD1. Thus, an increased capacity to modulate the expression of antioxidant proteins involved in RONS handling and calcium homeostasis may be critical for performance during high-intensity exercise in humans.

## Abbreviations

ATP: adenosine triphosphate
ATP5A: subunit of mitochondrial ATP synthase
BSA: bovine serum albumin
Ca^2+^: calcium ion
COXII: cytochrome *c* oxidase subunit II
CP: constant power
CSQ1: calsequestrin 1
CSQ2: calsequestrin 2
DEXA: dual-energy x-ray absorptiometry
FCR: free circulation recovery
F_I_O_2_: inspired oxygen fraction
HRmax: maximal heart rate
HRP: horseradish peroxidase
ISR: ischaemic recovery
Keap1: kelch-like ECH-associated protein 1
LLM: lower extremities lean mass
MAOD: maximal accumulated oxygen deficit
MCAv: middle-cerebral artery velocity
MFR: maximal functional reserve
MHC: myosin heavy chain
MHC I: myosin heavy chain type I
MHC IIa: myosin heavy chain type IIa
MHC IIx: myosin heavy chain type IIx
NDUFB8: NADH dehydrogenase [ubiquinone] 1 beta subcomplex subunit 8
NF-κB p65: nuclear factor NF-kappa-B p65 subunit
NIRS: near-infrared spectroscopy
Nrf2: nuclear factor (erythroid-derived 2)-like 2
NOX: NADPH oxidase
P38 MAPK: p38 mitogen-activated protein kinase
PCr: phosphocreatine
PFKM: phosphofructokinase
P_I_O_2_: partial pressure of inspired O_2_
PVDF: polyvinylidene fluoride
RER: respiratory exchange ratio
RONS: reactive oxygen and nitrogen species
ROS: reactive oxygen species
Rpm: revolutions per minute
SDHB: NADH dehydrogenase [ubiquinone] 1 beta subcomplex subunit 8
SERCA: sarco-endoplasmic reticulum Ca^2+^ ATPase
SODs: superoxide dismutase
TOI: tissue oxygenation index
TOI_MIN_: minimal 1-s rolling average TOI value during ischaemia
TOI_OBV_: mean TOI value registered during exercise
UQCRC2: ubiquinol-cytochrome-c reductase complex core protein 2
V_E_: ventilation
VO_2_: oxygen uptake
VO_2_max: maximal oxygen uptake
W: watts
Wmax: peak power output during the incremental exercise test to exhaustion
Wmean: mean power output during the Wingate test
Wpeak1-s: peak power output in 1-s averages during the Wingate test
Wpeak: instantaneous peak power output during the Wingate test
XO: xanthine oxidase

## 1 Introduction

During high-intensity exercise reactive oxygen and nitrogen species (RONS) production is exacerbated due to high oxidative and substrate level phosphorylation rates, and acidification caused by high glycolytic rates [[1], [2], [3], [4]]. While RONS have been implicated in muscle fatigue [1,[3], [4], [5]], exercise training reduces RONS production and enhances the antioxidant capacity of skeletal muscle by mechanisms mediated by the nuclear factor erythroid-derived 2-like 2 (Nrf2) [[6], [7], [8]]. Nrf2 activity depends on the Kelch-like ECH-associated protein 1 (Keap1) levels, which under unstressed conditions, acts as an adaptor for a ubiquitin E3 ligase complex, which tags Nrf2 for proteasomal degradation [9,10]. Keap1 is a redox sensor protein that, upon oxidative/electrophilic stress, stabilizes the Keap1-Nrf2 complex, impeding Nrf2 degradation [10]. When the level of free Keap1 is low, newly synthesized Nrf2 can translocate to the nucleus, where it interacts with antioxidant response elements (AREs), DNA sequences present in genes not only involved in the antioxidant response, but also in mitochondrial biogenesis, and metabolism, as well as other functions [9,10]. Interestingly, disruption of Keap1 in mice has been associated with enhanced exercise endurance [11].

Nevertheless, most studies indicate that acute oral administration of antioxidants to humans does not improve high-intensity exercise performance [12,13]. This contrasts with animal studies and *in vitro* experiments showing that antioxidants counteract fatigue with a dose-response curve that is ”U” shaped, i.e., an excessive amount of antioxidants appears to be detrimental [14]. The apparent insensitivity of human skeletal muscle to antioxidant administration may depend on the dose and type of antioxidant, the characteristics of the subjects and type of exercise employed. Thus, a novel exercise paradigm using repeated cycles of exercise to exhaustion followed immediately by a brief occlusion of the circulation and resumption of exercise with open circulation to elicit repeated cycles of ischaemia-reperfusion has been employed to investigate mechanisms of functional reserve [[15], [16], [17], [18], [19]]. This protocol can be used to test whether the characteristics of the subjects and their muscle phenotype, including resting expression of Keap1 and Nrf2, could explain differences in exercise performance and metabolism in humans repeatedly exercising to exhaustion.

During whole-body exercise at supramaximal exercise intensity, i.e., above the intensity eliciting VO_2_max, part of the energy required to supply the ATP demand must be provided by substrate level phosphorylation leading to accumulation of metabolites, fatigue, and task failure [20,21]. Nevertheless, part of the exercise capacity can be recovered after a few seconds or minutes of rest allowing the repetition of several supramaximal exercise bouts, although the work that can be produced in successive repetitions is progressively reduced [22,23]. This quick partial recovery of exercise capacity after task failure has been explained by a rapid resynthesis of phosphocreatine (PCr) [[24], [25], [26], [27]] and proton efflux allowing intracellular pH recovery [25,[28], [29], [30]], although intracellular pH recovery requires several minutes [29]. PCr resynthesis depends on O_2_ availability [29,31] and no PCr recovery is observed if ischaemia is applied at exhaustion [15,32] or if exercise is performed during ischaemia and the occlusion remains in place until exhaustion [33]. However, we have shown that a partial recovery of exercise capacity is possible despite the application of ischaemia at the end of high-intensity exercise to exhaustion [[15], [16], [17], [18], [19]]. Thus, some “functional reserve” or capacity to produce power at the same level or higher than reached at exhaustion exists at task failure, which depends on the glycolytic component of substrate level phosphorylation [15,16].

In previous studies, this “functional reserve” was observed after a single exercise bout to exhaustion [15], as well as after two bouts of exercise at 120% of VO_2_max [16]. This “functional reserve” should have a finite magnitude. However, in preceding experiments it was not possible to determine its actual value since the thirty-six subjects tested were able to show some functional reserve after two bouts of supramaximal exercise at 120% of VO_2_max [16]. It remains unknown how many bouts could be performed, and which factors may determine the magnitude of the “functional reserve”.

Therefore, the primary aim of this investigation was to determine the magnitude of the “functional reserve” using repeated bouts of supramaximal exercise interspaced with 20s recovery periods with occlusion of the circulation until incapability to re-start exercising. Additionally, we aimed to determine what mechanisms could explain the magnitude of the functional reserve. We hypothesized that a higher proportion of glycolytic enzymes, such as phosphofructokinase (PFKM), and a higher percentage of myosin heavy chain type II (MHC II) would associate with a higher functional reserve. Fatigue and task failure during high-intensity exercise has been also attributed to the effect of RONS, which may inactivate critical enzymes and interfere with calcium transients and calcium reuptake [[1], [2], [3], [4],^34^]. Therefore, we hypothesized that some proteins involved in calcium homeostasis like the sarco-endoplasmic reticulum Ca^2+^ ATPase (SERCAs) and calsequestrin 1 and 2 (CSQ1 and CSQ2, respectively), as well as the more abundant antioxidant enzymes in skeletal muscle (i.e., catalase and SODs) could play a role as determinants of the “functional reserve”.

Insufficient muscle and brain oxygenation can contribute to fatigue, and particularly a lower brain oxygenation during exercise performed until exhaustion has been associated with fatigue in several studies [[35], [36], [37], [38]]. Thus, we also hypothesized that the “functional reserve” may be greater in the subjects experiencing a lesser reduction in brain oxygenation during high-intensity exercise.

Thus, to achieve these aims we have conceived a new exercise protocol which allows for testing the response of skeletal muscle to repeated episodes of ischaemia-reperfusion under conditions of extreme fatigue in humans.

## 2 Materials and methods

### 2.1 Subjects

Thirty males and 13 females, all healthy and physically active agreed to participate in this investigation (Table 1). The inclusion criteria for participation in this investigation were: aged 18–35 years old; non-smoking; normal resting electrocardiogram, no chronic diseases or recent surgery; body mass index between 18 and 30 kg·m^−2^; no medical contraindications to exercise and no history of disease requiring medical treatments lasting longer than 15 days during the preceding six months. Subjects were requested to avoid strenuous exercise 48 h before the laboratory test and not to drink carbonated, caffeinated and alcohol-containing beverages during the 24 h preceding all tests. During the study period, subjects were also requested to abstain from the consumption of drugs, medications, and any dietary supplements. Sex and gender of the participants were defined based on self-report during participant recruitment. All participants reported *cis*-gender, and thereafter the terms males and females were applied in the study analysis and reporting [39]. All females were eumenorrheic, without taking oral contraceptives and were evaluated randomly in different phases of the menstrual cycle [40]. This approach is based on the similar sprint and high-intensity exercise responses observed in different phases of the menstrual cycle [[41], [42], [43]].

**Table 1:** Physical characteristics and ergoespirometric variables (mean ± SD).

	Males (n = 30)	Females (n = 13)	*p*
Age (years)	22.5 ± 2.1	22.2 ± 1.5	0.624
Height (cm)	176.7 ± 7.6	162.2 ± 4.5	**0.000**
Weight (kg)	73.3 ± 7.5	58.5 ± 11.6	**0.000**
% body fat	19.3 ± 5.1	27.9 ± 4.2	**0.000**
Legs lean mass (kg)	20.2 ± 1.7	13.9 ± 2.3	**0.000**
HRmax (Beats·min^−1^)	193.2 ± 8.3	195.1 ± 7.9	0.475
VO_2_max (mL·kg^−1^·min^−1^)	46.3 ± 6.4	39.9 ± 3.5	0.002
VO_2_max (mL·kg LLM^−1^·min^−1^)	166.7 ± 17.1	166.1 ± 15.6	0.913
Wmax (W)	263.2 ± 36.0	188.7 ± 40.0	**0.000**
MHC I	34.0 ± 12.4	53.5 ± 11.4	**0.000**
MHC IIa	51.2 ± 9.2	36.3 ± 6.5	**0.000**
MHC IIx	14.9 ± 9.6	10.2 ± 6.3	0.120
MHC I + IIa	85.2 ± 9.7	89.8 ± 6.3	0.123
120% VO_2_max CI (first bout, best performance)			
Time to exhaustion (s)	117.6 ± 39.2	131.9 ± 40.3	0.280
Power (W)	322.8 ± 49.3	219.4 ± 42.6	**0.000**
Power (W·kg LLM^−1^)	16.0 ± 2.2	15.7 ± 1.4	0.691
Work (kJ·kg LLM^−1^)	1.84 ± 0.50	2.05 ± 0.59	0.218
VO_2_ (mL·kg LLM^−1^·min^−1^)	129.1 ± 16.0	132.1 ± 13.3	0.561
O_2_/Work (mL·kJ- [1])	133.5 ± 19.5	140.1 ± 18.2	0.304
O_2_ demand (mL)	7806 ± 2867	6012 ± 1803	**0.044**
Accumulated O_2_ (mL)	5113 ± 2225	3986 ± 1369	0.099
O_2_ deficit (mL)	2692 ± 937	2026 ± 689	**0.026**
O_2_ deficit (mL·kg^−1^ BW)	36.6 ± 11.4	35.1 ± 11.6	0.690
O_2_ deficit (mL·kg LLM^−1^)	132.4 ± 40.7	145.5 ± 42.3	0.343
% Anaerobic Energy	35.4 ± 6.9	34.2 ± 7.8	0.604
RER	1.18 ± 0.11	1.08 ± 0.11	**0.009**
V_E_ (L·min^−1^)	89.5 ± 17.1	68.4 ± 12.6	**0.000**
RR (Breaths·min^−1^)	40.0 ± 6.6	41.7 ± 6.7	0.442
V_E_/ VO_2_	33.5 ± 5.5	34.6 ± 5.1	0.547
V_E_/ VCO_2_	29.8 ± 3.7	32.8 ± 5.4	**0.039**
P_ET_O_2_ (mmHg)	110.2 ± 4.7	111.8 ± 5.0	0.322
P_ET_CO_2_ (mmHg)	39.6 ± 4.5	34.2 ± 5.2	**0.001**
Vastus Lateralis TOI (a.u.)	62.2 ± 17.4	66.6 ± 4.9	0.381
Frontal lobe TOI (a.u.)	68.5 ± 5.5	63.4 ± 7.1	**0.016**
MCAv (mm·s^−1^)	44.7 ± 26.6	30.5 ± 35.1	0.153

HRmax: maximal heart rate; VO_2max_: maximal oxygen uptake; Wmax: maximal intensity during the incremental exercise test to exhaustion; LLM: lean mass of the lower extremities; Accumulated O_2_: total amount of O_2_ consumed during the test; and the average values during the 120% CP test for: RER, respiratory exchange ratio; V_E_, pulmonary ventilation; RR, respiratory rate; P_ET_O_2_, end tidal O_2_ pressure; P_ET_CO_2_, end tidal CO_2_ pressure; TOI, tissue oxygenation index; MCAv, middle cerebral artery velocity. Note: the supramaximal test data correspond to the session with the best performance time. P values based on two-tailed unpaired t-tests.

The study was approved by the Ethical Committee of the University of Las Palmas de Gran Canaria (Ref.: CEIH-2017-13) and performed in accordance with the standards set by the latest revision of the Declaration of Helsinki, except for registration in a database. All subjects signed a written informed consent before the start of the study.

### 2.2 Study overview

**Fig. 1:**
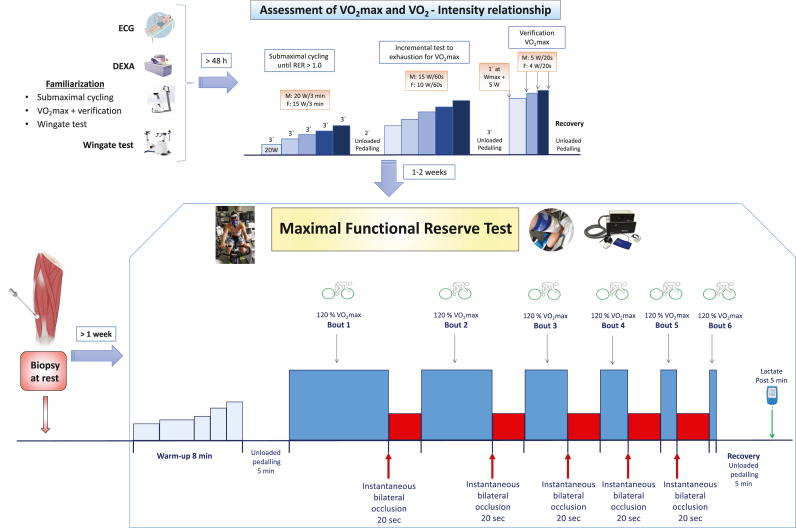
**Schematic representation of the study phases and exercise protocols.** Forty-three physically active participants (30 males and 13 females) were recruited for the study. After fulfilling inclusion criteria, a DEXA scan was performed after a 12-h overnight fast. On another visit, participants performed a familiarization session with the cycle ergometer exercise protocols that included submaximal cycling exercise, an incremental test until exhaustion with verification, a 30-s Wingate test and post-exercise occlusion of the circulation. On a third visit, participants carried out a test to assess VO_2_max and the VO_2_/intensity relationship. At least one week apart, the subjects were submitted to a resting muscle biopsy, which was separated by a minimum of one week from the performance of the maximal functional reserve tests (MFR). The MFR sessions consisted in two experimental sessions. In each of them, after a standardized warm-up, subjects performed six bouts of supramaximal constant intensity exercise at 120% of VO_2_max until exhaustion, interspersed either with 20 s of recovery periods with application of immediate post-exercise ischaemia at exhaustion (ischaemic recovery session) or with 20 s of recovery with free circulation (free circulation recovery session), in random order. At the start of the 2nd to 6th bouts in the ischaemic recovery session, the cuffs were deflated instantaneously, to allow for restoration of the circulation during the subsequent bout. The cuffs located around the two thighs were instantaneously inflated at 300 mmHg during the sessions with ischaemic recovery to elicit total occlusion of the circulation of both lower extremities and impede metabolic recovery. The schematic presented for the MFR test corresponds to the ischemic session. In 13 volunteers a muscle biopsy was obtained before and immediately after the ISR test.

The study included the following phases: a) familiarization and pre-testing; b) assessment of VO_2_max and the relationship between VO_2_ and exercise intensity; c) Wingate test, d) supramaximal exercise tests to determine the functional reserve at exhaustion; and e) obtaining muscle biopsies (Fig. 1).

### 2.3 Familiarization and pre-testing

Volunteers reported to the laboratory after a 12-h overnight fast for the assessment of their body composition by dual-energy x-ray absorptiometry (Lunar iDXA, GE Healthcare, Milwaukee, WI, USA) [44]. On a different visit, subjects were familiarized with the experimental procedures which included an incremental exercise to exhaustion with verification, sprint exercise (30-s Wingate all-out test) and post-exercise occlusion of the circulation. After that, subjects reported to the laboratory to complete different experimental tests on separate days.

### 2.4 Assessment of VO_2_max and the VO_2_/intensity relationship

The VO_2_max, maximal heart rate (HRmax), and maximal power output (Wmax) were determined in normoxia (F_I_O_2_: 0.21, P_I_O_2_: 144 mmHg) with an incremental exercise test until exhaustion with verification [45]. For this test, subjects reported to the laboratory at least 4 h after the last ingestion of food. The test started with 3 min at 20 W, followed by 15 W and 20 W increases every 3 min in females and males, respectively, until the respiratory exchange ratio (RER) was ≥1.00. After that, the load was increased by 10 W and 15 W every minute, in females and males, respectively, until exhaustion. The highest intensity attained in the test was taken as the Wmax of the incremental exercise. At exhaustion, the ergometer was unloaded, and slow pedalling (30–40 rpm) continued for 3 min. At the third minute of active recovery, the verification test was initiated at Wmax +5 W during 1 min, followed by a 4 and 5 W increase (females and males, respectively) every 20 s until exhaustion.

Oxygen uptake (VO_2_) was assessed by indirect calorimetry with a metabolic cart (Vyntus, Jaeger-CareFusion, Höchberg, Germany) operated in breath-by-breath mode. The gas analysers were calibrated immediately before each test using room air (20.93% O_2_ and 0.05% CO_2_) and high-grade certified gases provided by the manufacturer containing 16% O_2_ and 5% CO_2_. The volume flow sensor was calibrated at low (0.2 L/s) and high (2 L/s) rates immediately before each test. The validity of this metabolic cart was established by a butane combustion test [46], and its reliability checked during submaximal and maximal exercise intensities [47]. Respiratory variables were analysed breath-by-breath and averaged every 20 s during the incremental exercise tests. The highest 20-s averaged VO_2_ recorded during either the incremental test including the verification phase or the supramaximal exercise tests was taken as the VO_2_max [47]. Heart rate (HR) was recorded continuously with a sampling frequency of 1 s during all exercise tests via short-range radiotelemetry (RS400 and RS800, Polar Electro, Woodbury, NY, USA).

### 2.5 Wingate test

On a different day subjects reported to the laboratory to perform a 30-s Wingate test on the cycle ergometer set on isokinetic mode at 80 rpm (Lode Excalibur, Groningen, The Netherlands). Prior to the Wingate test a standardized warm-up was performed with 1 min of unloaded pedalling, followed by 2 min at 40 W or 60 W, 3 min at 60 or 80 W, 1 min at 80 or 100 W, 1 min at 100 or 120 W and 1 min at 120 or 140 W for females and males, respectively. This was followed by 5 min of unloaded pedalling at low cadence (20–40 rpm) and then the Wingate test was started. The data were analysed to obtain instantaneous peak power output (Wpeak), the peak power output in 1-s averages (Wpeak1-s) and the mean power output (Wmean).

### 2.6 Assessment of the functional reserve at exhaustion (maximal functional reserve tests)

The functional reserve at exhaustion was determined 1–2 weeks after the assessment of VO_2_max as previously reported [16]. From the total number of volunteers (n = 43), twenty-four males and six females reported to the laboratory on two occasions, one or two weeks apart, hereafter called ischaemic (ISR) and free circulation (FCR) recovery sessions (Fig. 1), while another group of 6 males and 7 females performed only the ISR protocol. The ISR session was utilized to determine the MFR, while the FCR was used as a control. The tests were performed approximately at the same time in both conditions, and subjects were requested to record the last meal ingested and reproduce it thoroughly before both experimental sessions. For those tests performed in the morning, subjects were requested to ingest a light breakfast, which should have been ended at least 1 h prior to the scheduled time for arrival to the laboratory. For those tests performed in the afternoon, only light meals ingested at least 4 h before the test were permitted. One hour after their arrival to the laboratory, the protocol described in Fig. 1 as “Maximal Functional Reserve Test” (MFR) started.

The ISR and FCR sessions were planned to include six bouts of constant-power exercise to exhaustion interspaced by 20 s recovery periods with (ISR) or without (FCR) application of total occlusion of the circulation to the lower extremities. The intensity of the supramaximal exercise bouts was set at 120% of VO_2_max, to facilitate the attainment of the maximal accumulated oxygen deficit during the first bout of constant-power exercise on the cycle ergometer [48,49]. For all tests, volunteers were instructed to maintain the pedalling rate steady at 80 rpm (±3 rpm) and remain seated on the cycle ergometer, i.e., no standing pedalling was permitted. The ergometer seat and handlebar configuration were adjusted for comfort during the first visit and replicated in subsequent sessions. In all instances, exhaustion was defined by the incapacity to maintain a pedalling cadence above 50 rpm during 5 s or by the sudden stop of pedalling. Strong verbal encouragement was provided for the continuation of the exercise, particularly if pedalling rate was declining or task failure imminent.

In the ISR session, the circulation of both lower extremities was instantaneously occluded at exhaustion after each bout. Right at the restart of the second and successive bouts, the cuffs were instantaneously released and the circulation fully re-established. If a given subject was not able to start exercising at 120% VO_2_max, the occlusion was instantaneously re-instated for another 20 s, while power was set at 100% of VO_2_max. Thereafter the bouts were repeated at 100% of VO_2_max until reaching the sixth bout or until incapacity to re-start pedalling. For those subjects performing only the ISR protocol, the sixth bout was followed by an additional occlusion of the circulation of both legs and a muscle biopsy was taken ∼10s after exhaustion with the occlusion in place.

The same approach was followed during the FCR sessions, but with the subjects performing unloaded pedalling (∼20 rpm) during the 20 s recovery periods, to minimize the risk of orthostatic post-exercise hypotension [50]. In the 20 s recovery period, after 15 s the subjects were given a 5 s reverse countdown and prompted to restart pedalling as fast as possible until the pedal cadence surpassed 80 rpm, which was the target pedalling rate for all bouts. After the last bout of the ISR and FCR tests, the subjects rested in the supine position and in 5 min a 5 μL blood sample was obtained from the hyperaemized earlobe to measure the capillary blood lactate concentration (Lactate Pro 2, Arkray Inc., Kyoto, Japan).

Before the exercise, while the subjects were resting supine, bilateral 10 cm wide cuffs were placed around the thighs, as close as possible to the inguinal crease, and connected to a rapid cuff inflator (SCD10, Hokanson E20 AG101, Bellevue, WA, USA) as previously reported [15,51]. Each session started with a warm-up consisting of 1 min of unloaded pedalling, followed by 2 min at 60 or 40 W, 3 min at 80 or 60 W, 1 min at 100 or 80 W, 1 min at 120 or 100 W and 1 min at 140 or 120 W for males and females, respectively. This was followed by 5 min of unloaded pedalling at low cadence (20–40 rpm). Then the subjects stopped pedalling, and the ergometer was set in hyperbolic mode at the load corresponding to their 120% of VO_2_max to perform the MFR test.

Cerebral and muscular oxygenation were assessed using near-infrared spectroscopy (NIRS, NIRO-200NX, Hamamatsu Photonics, Japan) employing spatially-resolved spectroscopy to obtain the tissue oxygenation index (TOI) using a path-length factor of 5.92 [52]. One NIRS optode was placed on the right frontoparietal region at 3 cm from the midline and 2–3 cm above the supraorbital crest, to avoid the sagittal and frontal sinus areas [50]. A second optode was placed in the lateral aspect of the thigh at middle length between the patella and the anterosuperior iliac crest, over the middle portion of the m. *vastus lateralis*. The *vastus lateralis* fractional extraction index (TOI O_2_ extraction index) was obtained as TOI_OBV_ - TOI_MIN_, where TOI_OBV_ is the mean TOI value registered during exercise and TOI_MIN_ is the minimal 1-s rolling average TOI value registered during ischaemia, as previously reported [16]. The validity of the TOI O_2_ extraction index has been shown by direct measurement of O_2_ extraction by measuring arterial and venous femoral O_2_ content during exercise in males and females [16]. The mean blood flow velocity in the middle-cerebral artery velocity (MCAv) was measured as an estimate of cerebral blood flow using two Doppler 2 MHz transducers applied bilaterally over the middle transtemporal window, as previously described [50].

The experiments were performed in an air-conditioned laboratory with an ambient temperature of ∼21 °C, a relative humidity of 60–80%, and ∼735 mmHg atmospheric pressure. All exercise tests were carried out on the same cycle ergometer (Lode Corival, Lode BV, Groningen, The Netherlands), which maintains the exercise intensity constant despite variations in pedalling rate.

The O_2_ demand during the supramaximal exercise bouts was estimated from the linear relationship between the last minute averaged VO_2_ of each load, from 20 to 40 W to the highest intensity with an RER <1.00. The accumulated oxygen deficit (AOD, an estimate of the energy provided by substrate-level phosphorylation), representing the difference between O_2_ demand and accumulated O_2_, was determined as previously reported [15,16,49]. The AOD in the first best bout was taken as the maximal accumulated oxygen deficit (MAOD) [53,54]. The contribution of anaerobic energy metabolism to the total energy yield was calculated as MAOD x 100/O_2_ demand. Since during the occlusions the myoglobin O_2_ stores are depleted and PCr is not resynthesized [15,32,55,56], the totality of the O_2_ deficit measured during the second and subsequent bouts in the ISR sessions corresponded to the energy supplied by the glycolytic component of substrate-level phosphorylation. The MFR was calculated as the sum of O_2_ deficits incurred from the second to the sixth bout, or the last exercise bout performed at 100% of VO_2_max. The total O_2_ deficit was calculated by adding the MFR to the MAOD.

### 2.7 Muscle biopsies

For biopsy sampling, volunteers reported to the laboratory at 07.00 h, following a 12-h overnight fast and a resting muscle biopsy was obtained from the middle portion of the m. *vastus lateralis* of one of the two thighs, assigned randomly, using the Bergstrom's technique with suction, as previously reported [15]. From the 13 participants (6 males and 7 females) performing only the ISR session, signalling responses Pre and Post-MFR tests were assessed in 12, due to one post-MFR missing value in one male participant. For this purpose, one biopsy was performed approximately ∼10 min before initiating the MFR test and immediately after the sixth bout from the same leg during ischaemia. The needle was directed distally with a 45° inclination for the Pre-MFR biopsy, and the skin incision was covered with a provisional plaster that allows a rapid removal at exhaustion for fast collection of the Post MFR biopsy. All biopsies were immediately frozen in liquid nitrogen and stored at – 80 °C until analysed.

### 2.8 Protein extraction and western blotting

Whole skeletal muscle lysates were prepared as previously reported [57], and total protein concentration quantified using the bicinchoninic acid assay [58]. Briefly, approximately 10 mg of muscle were homogenized in urea lysis buffer (6 M urea, 1% SDS), 50X Complete protease inhibitor and 10X PhosStop phosphatase inhibitor cocktails (Roche). Subsequently, the lysate was centrifuged for 12 min at 25,200 g at 16 °C. The resulting supernatant containing the protein fraction was diluted with electrophoresis loading buffer (160 mM Tris-HCl, pH 6.8, 5.9% SDS, 25.5% glycerol, 15% β-mercaptoethanol- bromophenol blue). The optimal amount of total protein from experimental samples to be loaded and the antibody concentrations for each assay were first determined by loading a gradient of control protein extracts (non-interventional human muscle prepared similarly as the experimental samples) in different amounts ranging from 1 to 35 μg. After confirming linearity within this range, equal amounts of protein of each sample (1.5–15 μg) were loaded and electrophoresed with SDS-PAGE using the system of Laemmli [59] and proteins were transferred onto the polyvinylidene fluoride (PVDF) membranes for protein blotting (Bio-Rad Laboratories, Hercules, CA, USA). To compensate for variability between gels, the samples from each subject were run onto the same gel together with an equal protein amount from an internal control (same as during linearity optimization) loaded in triplicate or quadruplicate. The densitometric value of the protein of interest was normalized to the mean value of the control sample.

Membranes were blocked for 1 h in in either 4% bovine serum albumin or 2.5–5% non-fat dried milk powder (blotting-grade blocker) diluted in Tris-buffered saline containing 0.1% Tween 20 (TBS-T) (BSA or Blotto blocking buffer) and incubated overnight at 4 °C with primary antibodies. Antibodies were diluted in 4% BSA-blocking buffer or 2.5–5% Blotto-blocking buffer. After incubation with primary antibodies, the membranes were washed and incubated for 1 h at room temperature with an HRP-conjugated anti-rabbit or anti-mouse antibody (diluted 1:5000 to 1:20000 in 5% Blotto blocking buffer in all instances) and subsequent chemiluminescent visualization with Clarity™ Western ECL Substrate (Bio-Rad Laboratories) using the ChemiDocTM Touch Imaging System (Bio-Rad Laboratories). Densitometry band quantification was performed with the Image Lab© software 5.2.1 (Bio-Rad Laboratories). Equal loading and transfer efficiency was verified by staining the membranes with Reactive Brown 10 (Sigma-Aldrich, St. Louis, MO, USA). The corresponding catalogue numbers of primary antibodies were as follows: pSer^40^ Nrf2 (no. ab76026), Nrf2 (no. ab62352), SOD1 (no. ab16831) and the anti-OXPHOS premixed cocktail antibody (total OXPHOS human antibody cocktail, no. ab110411) purchased from Abcam (Cambridge, UK); catalase (no. 14097), SOD2 (no. 13141) and myoglobin (D2F5X, Rabbit mAb #25919), pThr^180^/Tyr^182^ p38 MAPK (no. CS9211), p38 MAPK (no. 9212), and pSer^536^ NFκB p65 (no. 3033), NFκB p65 (no. 3034) from Cell Signalling Technology (Danvers, MA, USA); calsequestrin1 (no. C0618), calsequestrin2, (no. 3868), SERCA1 (no. WH0000487M1) and SERCA2 (no. S1439) from Sigma-Aldrich and Keap1 (no. 10503-2-AP), phosphofructokinase1 (PFKM) (55028-1-AP) and citrate synthase (16131-1-AP) were purchased from Proteintech (Rosemont, IL, USA). The secondary HRP-conjugated goat anti-rabbit (no. 111-035-144) and the HRP-conjugated goat anti-mouse (no.115-035-003) antibodies were acquired from Jackson ImmunoResearch Inc. (West Grove, PA, USA). A more detailed description of materials and procedures used for western blotting is available in the Supplementary Table 1.

### 2.9 Myosin heavy chain analysis

Determination of MHC isoform proportions was performed on the muscle biopsies using sodium dodecyl sulphate polyacrylamide gel electrophoresis (SDS-PAGE). Experimental samples from the exact same aliquots as used for western blotting were loaded (7.5–10 μg) in triplicate onto the same gel, together with two internal control samples. The inclusion of two lanes loaded with the same internal control in all gels was used for quality check and accurate quantification of the variability of the assays, and not for normalization purposes. Experimental samples and controls were run at 4 °C on an SDS-PAGE gel containing a 3% acrylamide (v/v) phase (stacking gel) for ∼12 h at 70 V and afterwards on a 6% acrylamide (v/v) and 30% glycerol (v/v) phase (resolving gel) for ∼20 h at 350 V. Subsequently, the gels were Coomassie stained for ∼1 h followed by distaining with a 40% methanol (v/v) and 10% glacial acetic acid (v/v) solution for ∼ 1 h and lastly by background subtraction submerging the gel in distilled water for ∼1 h. Then, the MHC isoform content was determined by scanning the gel with a densitometry scanner (GS-800 Imaging Densitometer, Bio-Rad Laboratories, Hercules, CA, USA) and quantified with the Image Lab© software 5.2.1 (Bio-Rad Laboratories).

### 2.10 Statistics

The Gaussian distribution of variables was determined with the Shapiro-Wilks test, and when required, data were transformed logarithmically before further analysis. Muscle protein expression values in biopsies taken before and after exercise were compared using a paired *t*-test. Differences between males and females were determined using an unpaired *t*-test. Linear relationships between variables were examined by simple and multiple linear regression analyses. Unless otherwise stated, results are reported as the mean ± standard deviation (SD). Statistical significance was set at p < 0.05. Statistical analyses were performed using IBM SPSS Statistics v.21 for Mac (SPSS Inc., Chicago, IL, USA) and Jamovi v1.8.1. (Jamovi project, 2021).

## 3 Results

### 3.1 General physical characteristics and performance

The physical characteristics and performance during the incremental exercise to exhaustion and the best 120% constant-power first bout (120% CP) are reported in Table 1 for males and females. The performance achieved during the Wingate test is reported in Table 2. Males and females had comparable VO_2_max per kg of LLM (166.7 ± 17.1 and 166.1 ± 15.6 ml·kg LLM^−1^.min^−1^, P = 0.91) and had similar peak and mean power output per kg of LLM in the Wingate test (Table 2), as well as MAOD values normalized to LLM during the ISR test (Table 1). VO_2_max per kg of LLM was associated with the expression levels of the mitochondrial proteins COXII (r = 0.52, P < 0.001, n = 43), UQCRC2 (r = 0.33, P = 0.029, n = 43), ATP5A (r = 0.52, P < 0.001, n = 43), and NDUFB8 (r = 0.38, P = 0.019, n = 43). VO_2_max per kg of LLM was also associated with MHCI + IIa (r = 0.50, P < 0.001, n = 43) and SOD2 protein expression levels (r = 0.42, P = 0.006, n = 42).

**Table 2:** Wingate test performance (mean ± SD).

	Males (n = 29)	Females (n = 11)	*p*
Wpeaki (W)	1010 ± 206	707 ± 185	**0.000**
Wpeak1-s (W)	812 ± 149	564 ± 140	**0.000**
Wmean (W)	575 ± 95	381 ± 91	**0.000**
Wpeaki (W·kg BW^−1^)	13.8 ± 2.6	12.7 ± 3.0	0.245
Wpeak1-s (W·kg BW^−1^)	11.1 ± 2.0	10.1 ± 2.3	0.173
Wmean (W·kg BW^−1^)	7.9 ± 1.0	6.8 ± 1.5	**0.019**
Wpeaki (W·kg LM^−1^)	18.1 ± 3.3	18.2 ± 3.9	0.881
Wpeak1-s (W·kg LM^−1^)	11.1 ± 2.0	10.1 ± 2.3	0.173
Wmean (W·kg LM^−1^)	10.3 ± 1.3	9.9 ± 1.9	0.432
Wpeaki (W·kg LLM^−1^)	50.0 ± 9.2	51.8 ± 11.0	0.595
Wpeak1-s (W·kg LLM^−1^)	40.3 ± 6.6	41.5 ± 8.5	0.634
Wmean (W·kg LLM^−1^)	28.5 ± 3.5	28.1 ± 5.7	0.796

Wpeaki: instantaneous peak power output; Wpeak1-s: peak power output for 1-s averages; Wmean: mean power output. BW: body weight; LM: whole body lean mass; LLM: lean mass of the lower extremities. P values based on two-tailed unpaired t-tests. One male and one female did not perform the Wingate test.

### 3.2 Impact of the exercise protocol on p38 MAPK and NF-κB p65 phosphorylation as surrogate indicators of RONS-induced signalling

**Supplementary Fig. 1:**
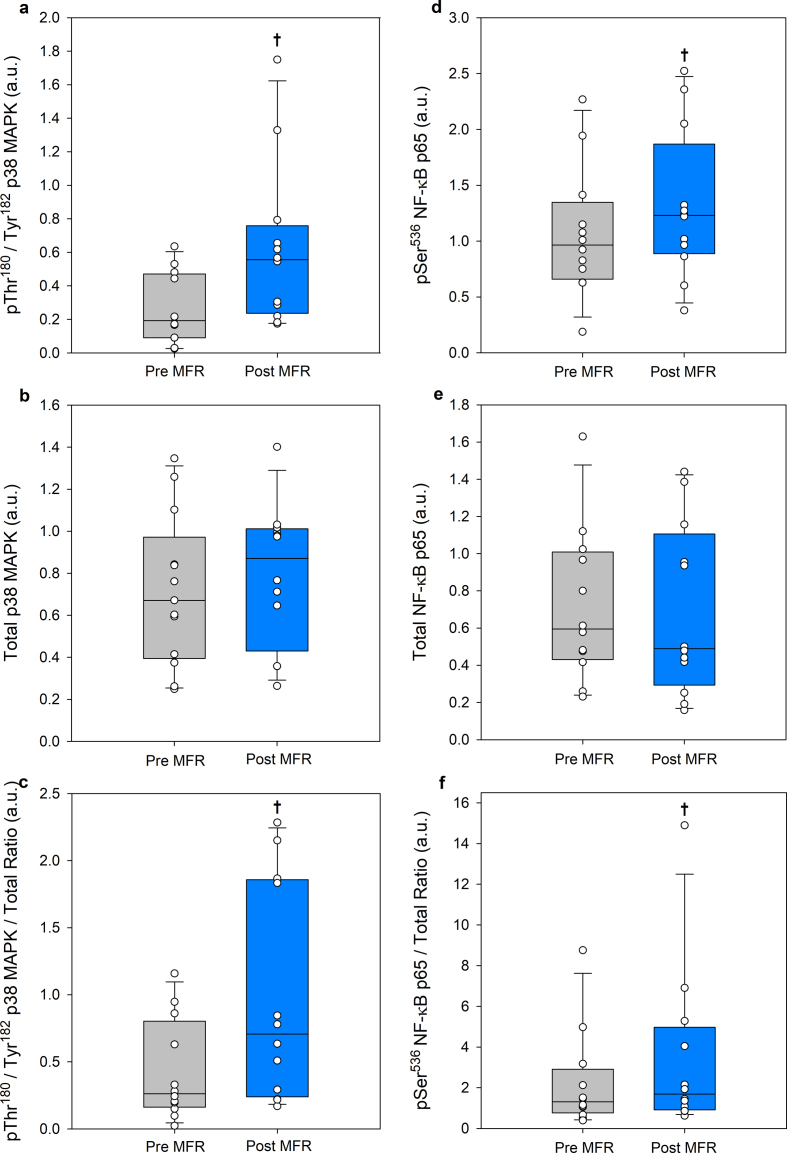
Responses of RONS-sensitive kinases to the ISR session. p38 mitogen-activated protein kinase (P38 MAPK) (a-c); Nuclear factor NF-kappa-B p65 subunit (NF-κB p65) (d-f), n = 12; 5 M and 7 F. Pre MFR, before the maximal functional reserve test; Post MFR, ∼10s following exhaustion in the maximal functional reserve test with the occlusion in place. The values shown are means ± standard errors and expressed in arbitrary units (a.u.). † P<0.05 compared to Pre MFR.

**Supplementary Fig. 2:**
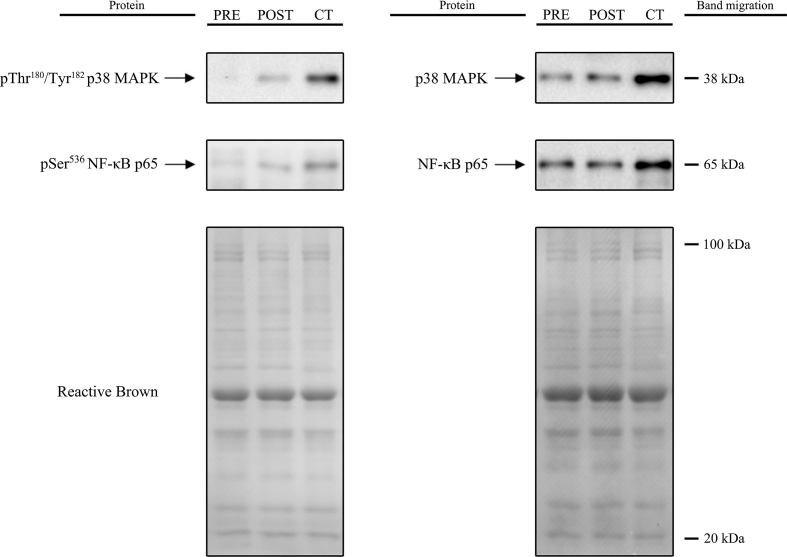
Representative immunoblot images and total amount of protein loaded (Reactive Brown Staining) for the proteins illustrated in Supplementary Figure 1, corresponding to one male participant. CT, a non-intervention human sample included on each gel as loading control. The standard markers closest to the band migration are indicated on the right side of the panel.

The ISR protocol substantially increased the phosphorylation of pThr^180^/Tyr^182^ p38 MAPK (P = 0.016, n = 12). Additionally, the ratio of phosphorylated pThr^180^/Tyr^182^ p38 MAPK to total p38 MAPK exhibited a notable elevation (P = 0.051, n = 12). The latter was accompanied by increased phosphorylation of pSer^536^ NF-κB p65 (P = 0.004, n = 12), along with an elevated ratio pSer^536^ NF-κB p65/NF-κB p65 (P = 0.005, n = 12), indicating activation of the RONS-sensitive NF-κB signalling pathway (Supplementary Figs. 1 and 2).

### 3.3 Maximal functional reserve

Both sexes showed a similar physiological response to the ISR test when differences in lean mass were accounted for (Table 3). The MFR expressed as total work was similar in males and females when normalized to LLM. This work was developed at a mean intensity above the Wmax in both sexes, with a similar contribution by the glycolytic component of substrate level phosphorylation. Twelve out of 30 males accomplished 6 bouts, i.e., 5 occlusions in the ISR test (8 performed the six bouts at 120% of VO_2_max, while 4 did the last bouts at 100% of VO_2_max). The rest of the males were able to perform 5 bouts (n = 12) or 4 bouts (n = 6), while 8 females performed 6 bouts (six performed the six bouts at 120% of VO_2_max, while two did 4 at 120% and 2 at 100% of VO_2_max), 2 females performed 5 bouts, one 4 bouts, and 2 only 3 bouts. The tests with free circulation recovery were carried out by 24 males and 6 females. All subjects could perform the six bouts programmed, except for one female who did five bouts (three at 120% and two at 100% of VO_2_max). The total work performed was 67% higher in the test with free circulation during the recovery periods (FCR session) when the same number of bouts performed in the ISR tests were compared (P < 0.001). Subjects were able to perform more bouts and at a higher mean relative intensity when they recovered with free circulation.

**Table 3:** Ergospirometric variables assessed during the maximal functional reserve test (mean ± SD).

Maximal functional reserve (tests with ischaemic recovery)			
	Males (n = 30)	Females (n = 13)	*p*
Time to exhaustion (s)	77.6 ± 35.1	110.1 ± 69.2	0.129
Power (W)	290 ± 51	203 ± 48	**0.000**
Power (W·kg BW^−1^)	4.0 ± 0.7	3.5 ± 0.4	**0.020**
Power (W·kg LM^−1^)	5.2 ± 0.8	5.1 ± 0.6	0.768
Power (W·kg LLM^−1^)	14.4 ± 2.3	14.5 ± 1.9	0.853
Work (kJ)	22.3 ± 10.4	22.9 ± 15.9	0.899
Work (kJ·kg BW^−1^)	0.30 ± 0.13	0.39 ± 0.26	0.287
Work (kJ·kg LM^−1^)	0.40 ± 0.17	0.56 ± 0.36	**0.046**
Work (kJ·kg LLM^−1^)	1.10 ± 0.48	1.61 ± 1.01	0.598
O_2_ demand (mL)	4711 ± 2328	4730 ± 3595	0.984
Accumulated O_2_ (mL)	3248 ± 1821	3360 ± 2557	0.471
Accumulated O_2_ (mL·kg LLM^−1^)	160 ± 85	236 ± 164	0.674
O_2_ deficit (mL)	1463 ± 709	1370 ± 1097	0.746
O_2_ deficit (mL·kg BW^−1^)	19.9 ± 8.8	23.0 ± 16.5	0.545
O_2_ deficit (mL·kg LM^−1^)	26.1 ± 11.6	33.3 ± 23.3	0.184
O_2_ deficit (mL·kg LLM^−1^)	72.4 ± 32.2	95.4 ± 66.4	0.271
O_2_/Work (mL·kJ^−1^)	127 ± 28	143 ± 20	0.070
O_2_/Power (mL·W^−1^)	14.4 ± 7.0	17.8 ± 11.5	0.348
% Anaerobic Energy	40.2 ± 10.8	33.5 ± 9.8	0.071
RER	1.33 ± 0.11	1.26 ± 0.23	0.183
V_E_ (L·min^−1^)	114.4 ± 18.6	91.7 ± 17.7	**0.001**
RR (Breaths·min^−1^)	52.2 ± 8.4	57.7 ± 8.9	0.069
V_E_/ VO_2_	51.7 ± 8.6	52.8 ± 10.3	0.725
V_E_/ VCO_2_	35.9 ± 4.6	41.4 ± 6.1	**0.003**
P_ET_O_2_ (mmHg)	120.9 ± 3.4	121.2 ± 3.9	0.783
P_ET_CO_2_ (mmHg)	30.3 ± 3.7	28.2 ± 2.8	0.203
Vastus Lateralis TOI (%)	62.0 ± 3.9	62.3 ± 5.2	0.835
Frontal lobe TOI (%)	63.6 ± 6.1	58.1 ± 8.9	**0.030**
MCAv (cm.s^−1^)	40.6 ± 6.8	40.8 ± 14.6	0.966

HRmax: maximal heart rate; VO_2max_: maximal oxygen uptake; Wmax: maximal intensity during the incremental exercise test to exhaustion; LLM: lean mass of the lower extremities; Accumulated O_2_: total amount of O_2_ consumed during the test; and the average values during the 120% CP test for: RER, respiratory exchange ratio; $\dot{V}$_E_, pulmonary ventilation; RR, respiratory rate; P_ET_O_2_, end tidal O_2_ pressure; P_ET_CO_2_, end tidal CO_2_ pressure; TOI, tissue oxygenation index; MCAv, middle cerebral artery velocity. P values based on two-tailed unpaired t-tests.

**Supplementary Fig. 3:**
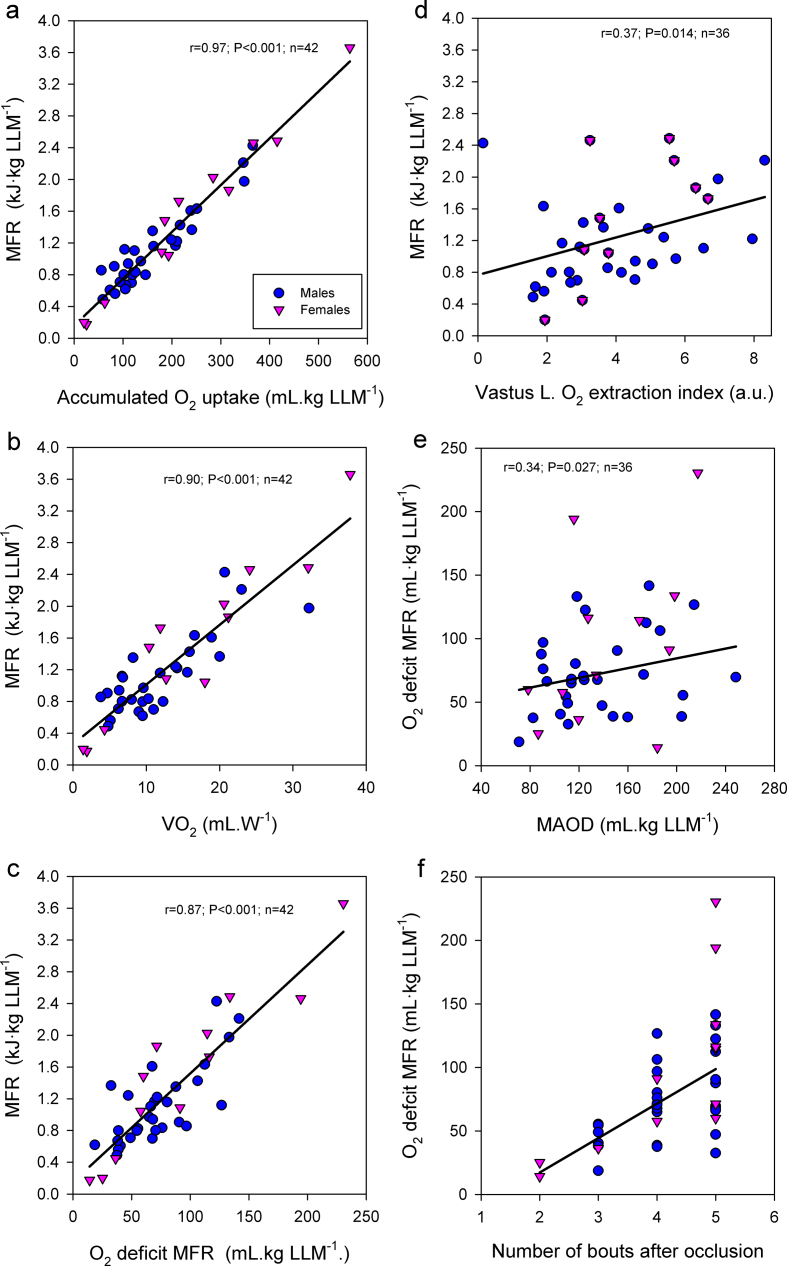
Associations between the maximal functional reserve (MFR) in kJ·kg LLM^−1^ and the accumulated O_2_ uptake (a), the O_2_ consumed per watt (b), the O_2_ deficit in the MFR test (c), and the vastus lateralis O_2_ fractional extraction index (d), as well as association between the O_2_ deficit in the MFR test with the maximal accumulated O_2_ deficit (MAOD) (e) and the number of bouts performed in the MFR test after the first occlusion (f). Each point represents an individual subject. Male, blue circles; Female, pink triangles. Correlation coefficients and regression lines have been calculated using the individual values. n = 43; 30 males and 13 females.

There were linear associations between the MFR in kJ·kg LLM^−1^ and the accumulated VO_2_ in mL·kg LLM^−1^ (r = 0.97, P < 0.001, n = 42) in the MFR test, the O_2_ consumed per watt in mL·W^−1^ (r = 0.90, P < 0.001, n = 42), the O_2_ deficit in mL·kg LLM^−1^ in the MFR test (r = 0.87, P < 0.001, n = 42), and the *vastus lateralis* O_2_ fractional extraction index (r = 0.37, P = 0.014, n = 36) (Supplementary Figs. 3a–d). The O_2_ deficit per kg of LLM in the MFR test was linearly associated with the MAOD (r = 0.34, P = 0.027, n = 43) and the number of bouts performed in the test (r = 0.58, P < 0.001, n = 42) (Supplementary Figs. 3e and f). The *vastus lateralis* O_2_ fractional extraction index was inversely associated with the Log of SOD1 protein expression (r = −0.50, P < 0.001, n = 42), Log of Nrf2 total protein expression (r = −0.38, P < 0.012, n = 43), and the Log of Nrf2/Keap1 (r = −0.34, P < 0.026, n = 43).

The power developed per kg of LLM in the functional reserve test was linearly associated with the VO_2_max in ml•kg LLM^−1^•min^−1^ (r = 0.45, P < 0.001, n = 43), the Wmean per kg of LLM in the Wingate test (r = 0.46, P = 0.003, n = 40), the percentage of MHC I (r = 0.31, P = 0.040, n = 43), and the protein expression of COXII (r = 0.31, P = 0.044, n = 43).

### 3.4 Brain perfusion and oxygenation

No association was observed between brain oxygenation and perfusion, as indicated by the frontal lobe TOI and the MCAv, and the MFR in kJ·kg LLM^−1^. No significant differences were observed in frontal lobe oxygenation between the ISR and FCR sessions (64.2 ± 5.6 and 65.7 ± 4.8 a.u., n = 25, P = 0.21).

### 3.5 The O_2_ deficit during the maximal functional reserve test is associated with some proteins involved in redox regulation

The O_2_ deficit per kg of LLM in the MFR test was linearly associated with the protein expression levels in the *vastus lateralis* of pSer^40^-Nrf2 (r = 0.48, P < 0.001, n = 43) and the Log of SOD1 (r = −0.34, P < 0.030, n = 42). Likewise, capillary blood lactate concentration 5 min after the end of the MFR test was linearly associated with V_E_ (r = 0.38, P = 0.014, n = 30), and protein expression of catalase (r = 0.38, P = 0.013, n = 30), myoglobin (r = 0.36, P = 0.017, n = 30) and the Log of CQS2 (r = 0.39, P = 0.011, n = 30). Keap1 was associated with the percentage of anaerobic energy yield during the MFR (r = 0.41, P = 0.008, n = 42).

### 3.6 Main predictors of maximal functional reserve

Multiple regression analysis indicated that the main variables predicting the MFR expressed as total work normalized to LLM were the accumulated O_2_ uptake per kg of LLM during the MFR test, which explained 94.1% of the variance; the O_2_ deficit per kg of LLM during the MFR test, which explained an additional 2.9% of the variance; the mean power output per kg of LLM during MFR test, which explained an additional 1.3% of the variance, and the percentage of MHC I + IIa which explained 0.3% of the variance (R^2^ = 0.985, P < 0.001, Table 4). The accumulated O_2_ uptake in the MFR test was predicted by the O_2_ deficit per kg of LLM, which explained 59.4% of the variance; SERCA1 protein expression, the frontal lobe oxygenation index, Keap1 protein expression, SERCA2 protein expression, and myoglobin protein expression accounted for 6.7, 6.1, 4.6, 3.2, and 2.8% of the variance, respectively (R^2^ = 0.83, P < 0.001, Table 4). The O_2_ deficit in the MFR test per LLM was predicted by the protein expression levels of pSer^40^-Nrf2, which explained 21.5% of the variance; the MAOD, which explained 14% of the variance, and the Log SOD1 protein expression levels, which explained 7.1% (R^2^ = 0.45, P = 0.001, Table 4). The mean power output in the MFR test expressed as W per kg of LLM was predicted by the aerobic efficiency in the MFR test expressed as mL of O_2_ per kJ, which explained 32.7% of the variance, the VO_2_max in mL·kg^−1^•min^−1^, which explained 20.1% of the variance, Keap1 expression levels 5.6%, and SERCA2 protein expression levels, which explained an additional 6.0% of the variance (R^2^ = 0.65, P < 0.001, Table 4).

**Table 4:** Predictive models for the maximal functional reserve (MFR).

**Predictive model for the maximal functional reserve (MFR) expressed as kJ per kg of lower extremities lean mass (LLM)**													
Predictor	Estimate	SE	95% Confidence Interval		t	p	Stand. Estimate	95% Confidence Interval		Model	Model fit measures		
			Lower	Upper				Lower	Upper		R	R^2^	P
Intercept	−0.18099	0.14754	−0.47993	0.11795	−1.23	0.228							
Accumulated VO_2_ (mL· kg LLM^−1^)	0.00503	0.000206	0.00461	0.00545	24.47	**< .001**	0.8226	0.7545	0.8908	1	0.97	0.941	**< .001**
O_2_ deficit (mL·kg LLM^−1^)	0.00295	0.000547	0.00185	0.00406	5.4	**< .001**	0.1871	0.1169	0.2572	2	0.985	0.97	**< .001**
Mean power in MFR test (w·kg LLM^−1^)	0.04553	0.00703	0.03129	0.05977	6.48	**< .001**	0.1425	0.0979	0.1871	3	0.992	0.983	**< .001**
MHC I + IIa (%)	−0.00451	0.00165	−0.00786	−0.00116	−2.73	**0.01**	−0.056	−0.0977	−0.0144	4	0.993	0.986	**< .001**

**Predictive model for the accumulated O**_**2**_**uptake during the MFR test expressed as mL per kg of lower extremities lean mass (LLM)**													
Intercept	555.8	111.992	327.09	784.52	4.96	**< .001**							
O_2_ deficit (mL·kg LLM^−1^)	1.77	0.211	1.33	2.2	8.36	**< .001**	0.685	0.5174	0.85211	1	0.771	0.594	**< .001**
Frontal lobe TOI (a.u.)	−5.3	1.566	−8.5	−2.1	−3.38	**0.002**	−0.289	−0.4635	−0.11455	2	0.81	0.655	**< .001**
SERCA1 protein expression (a.u.)	−57.53	25.052	−108.69	−6.37	−2.3	**0.029**	−0.183	−0.345	−0.02022	3	0.85	0.723	**< .001**
Keap1 protein expression (a.u.)	−54.93	25.98	−107.99	−1.87	−2.11	**0.043**	−0.174	−0.3418	−0.00592	4	0.877	0.769	**< .001**
SERCA2 protein expression (a.u.)	13.88	5.284	3.09	24.67	2.63	**0.013**	0.208	0.0464	0.37019	5	0.895	0.801	**< .001**
Myoglobin protein expression (a.u.)	−39.07	17.571	−74.96	−3.19	−2.22	**0.034**	−0.197	−0.3776	−0.01606	6	0.91	0.829	**< .001**

**Predictive model for the O**_**2**_**deficit during the MFR test expressed as mL per kg of lower extremities lean mass (LLM)**													
Intercept	−14.359	20.685	−56.271	27.553	−0.694	0.492							
pSer^40^Nrf2 protein expression (a.u.)	73.477	20.476	31.99	114.965	3.589	**< .001**	0.452	0.197	0.7077	1	0.484	0.234	**0.001**
MAOD (mL·kg LLM^−1^)	0.406	0.125	0.152	0.66	3.237	**0.003**	0.399	0.149	0.6494	2	0.612	0.374	**< .001**
Log SOD1 protein expression (a.u.)	−53.736	24.788	−103.961	−3.51	−2.168	**0.037**	−0.274	−0.53	−0.0179	3	0.667	0.445	**< .001**

**Predictive model for the mean power in MFR test expressed as W per kg of lower extremities lean mass (LLM)**													
Intercept	13.3272	2.66098	7.9355	18.7188	5.01	**< .001**							
O_2_ cost, O_2_/Work (mL·kJ^−1^)	−0.054	0.00838	−0.071	−0.037	−6.44	**< .001**	−0.652	−0.857	−0.4468	1	0.572	0.327	**< .001**
VO_2_max (mL·kg LLM^−1^)	0.0611	0.01293	0.0349	0.0873	4.72	**< .001**	0.457	0.2613	0.6536	2	0.727	0.528	**< .001**
Keap1 protein expression (a.u.)	−1.5425	0.56474	−2.6868	−0.3982	−2.73	**0.01**	−0.277	−0.4821	−0.0715	3	0.771	0.594	**< .001**
SERCA2 protein expression (a.u.)	0.3192	0.12625	0.0634	0.575	2.53	**0.016**	0.245	0.0486	0.4407	4	0.809	0.654	**< .001**

MAOD, maximal accumulated oxygen deficit; MCH, myosin heavy chain; Nrf2, nuclear factor (erythroid-derived 2)-like 2; SERCA, sarco-endoplasmic reticulum Ca^2+^ ATPase; Keap1, kelch-like ECH-associated protein 1; SOD, superoxide dismutase; Log, logarithm.

### 3.7 Sex differences in muscle phenotype

**Fig. 2:**
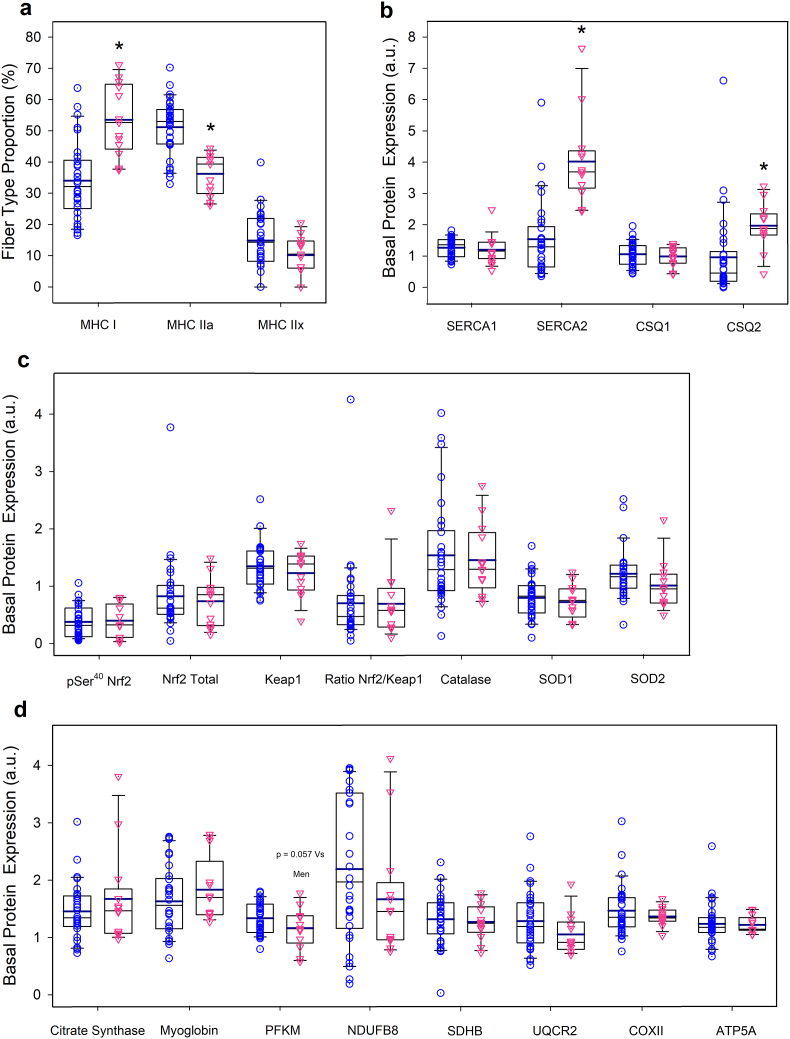
Sex differences in muscles in myosin heavy chain proportions (a), and basal expression of proteins involved in calcium handling (b), redox regulation (c), and muscle metabolism and O_2_ transport (d). n = 43; 30 M and 13 F, except for SOD1 and SOD2 with n = 42. Males: blue circles; females: red triangles. The values shown are means ± standard errors and expressed in arbitrary units (a.u.). * P < 0.05 for between-sex differences.

**Fig. 3:**
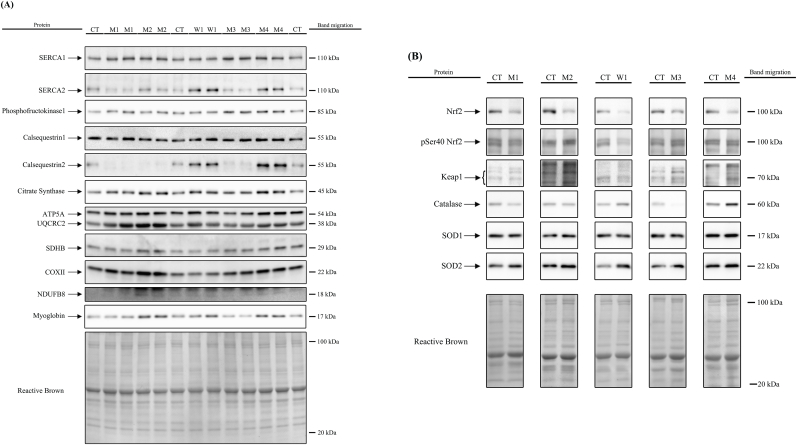
Representative images of basal protein expression levels (Western Blot) for all proteins studied and total amount of protein loaded (Reactive Brown Staining) from one woman and four men participating in the study. A control human sample (non-experimental) was included onto each gel in triplicate or quadruplicate to allow normalization and as a loading control. Images from top to bottom: SERCA1, SERCA2, phosphofructokinase1, calsequestrin1, calsequestrin2, citrate synthase, ATP5A, UQCRC2, SDHB, COXII, NDUFB8, myoglobin and Reactive Brown (as total protein loading control) with experimental samples run in duplicate (Panel A), and Nrf2, pSer^40^ Nrf2, Keap1, catalase, SOD1, SOD2 and Reactive Brown (Panel B). CON, control non-experimental sample; M, sample indicating a male participant; W, sample indicating a female subject. Estimated molecular weights are indicated on the right side of the blot.

Females had a higher percentage of MHC I (53.5 ± 11.4 vs. 34.0 ± 12.4%, P < 0.001) and higher basal expression of SERCA2 (4.0 ± 1.4 vs 1.5 ± 1.2 a.u., P < 0.001) and CSQ2 (1.0 ± 1.3 vs 2.0 ± 0.7 a.u., P = 0.014). Although PFKM was 15% higher in males than females, the difference did not reach statistical significance (P = 0.057) (Fig. 2, n = 13 and 30, for females and males, respectively, unless otherwise indicated in the figure legend). Representative immunoblots of all proteins studied are depicted in Fig. 3.

## 4 Discussion

The present investigation shows in humans that the protein expression of enzymes involved in both sensing and counteracting RONS likely play an important role in determining the exercise capacity during high-intensity exercise requiring a marked contribution of glycolysis for muscle energy production. We have demonstrated that the MFR has a finite magnitude ranging from 0.17 to 3.66 kJ·kg LLM^−1^ in healthy young humans, with comparable values in males and females. We have also shown that the MFR is associated with both the O_2_ consumed and the O_2_ deficit observed during the MFR test, indicating that both oxidative and substrate level phosphorylation contribute to this functional reserve. However, conceptually no functional reserve would exist without a metabolic reserve in glycolytic substrate level phosphorylation, since the second exercise bout was always performed at a work rate of 120% of VO_2_max. Moreover, even for the bouts carried out at 100% of VO_2_max a contribution from substrate level phosphorylation is required to re-start exercising, given the mismatch between O_2_ demand and VO_2_ at the beginning of exercise. Overall, these results imply that during repeated supramaximal exercise to task failure, a large metabolic reserve exists in both oxidative and substrate level phosphorylation at exhaustion, the latter been explained by its glycolytic component, as previously reported [15,16]. We have also shown that the aerobic component of the MFR is positively associated with the total O_2_ deficit and SERCA2 protein expression levels and negatively with the exercising frontal lobe oxygenation and the basal protein expression levels of SERCA1, Keap1, and myoglobin. In turn, the anaerobic component of the MFR is positively associated with the basal protein expression levels of pSer^40^-Nrf2 and the MAOD and negatively with the basal protein expression levels of SOD1. Finally, the mean power output developed during the MFR test was associated positively with the VO_2_max in mL•kg LLM^−1^•min^−1^ and SERCA2 protein expression levels, and negatively with the aerobic cost of exercise in mL of O_2__·_kJ^−1^ and Keap1 protein expression levels. Globally, these findings indicate that MFR test performance depends on the contribution of anaerobic capacity, which is essential, and the VO_2_max which establishes the mean power output that can be developed in the MFR test.

### 4.1 Is the functional reserve explained by muscle properties?

In agreement with our hypotheses, the ISR protocol elicited the phosphorylation of p38 MAPK and NF-κB p65, which depends on exercise-produced RONS [[60], [61], [62], [63]]. Accordingly, the functional reserve and its aerobic and glycolytic components can be predicted by variables intrinsically linked to the muscle phenotype, mostly related to redox regulation and calcium handling. As shown in Table 4, multiple regression analysis indicates that the percentage of MHC I + IIa contributes to predicting the MFR in kJ·kg LLM^−1^, however, the proportion of variance explained by MHCs is very small compared to the contribution made by the aerobic and the glycolytic components of the MFR, which explain 94 and 3% of the variance in MFR in kJ·kg LLM^−1^. Nonetheless, the mean power output developed during the MFR test was positively associated with indices of aerobic metabolism with the main role played by the O_2_ cost of exercise, which had a negative impact, meaning that the higher the O_2_ cost of exercise, the lower the MFR. In contrast, a higher VO_2_max predicts a greater power per kg of LLM during the MFR test. These two findings indicate that both the VO_2_max and the efficiency with which the O_2_ is utilized are crucial determinants of the power that can be developed during the MFR test. Thus, we decided to examine which factors could explain the aerobic and the anaerobic components of the functional reserve.

### 4.2 A higher functional reserve is mostly explained by a greater capacity to utilize O_2_ at exhaustion combined with an enhanced reserve in glycolytic capacity

The present results indicate that the aerobic component of the functional reserve depends on multiple factors defined by intrinsic properties of the exercising muscles like the glycolytic capacity to resynthesize ATP during exercise, the basal expression of the ROS-sensor protein Keap1, the basal protein expression levels of the calcium-regulating proteins SERCA1 and SERCA2, and the basal expression of myoglobin, as commented in the lines that follow. It should be highlighted that the main predictor of the aerobic component of the functional reserve is the O_2_ deficit, which represents the contribution to ATP resynthesis made by the glycolytic component of substrate-level phosphorylation. This is explained by the fact that the subjects with a higher O_2_ deficit were able to perform more bouts of exercise, i.e., the glycolytic component of the MFR determines the capacity to restart exercising after each occlusion.

Two isoforms of sarco/endoplasmic reticulum Ca^2+^-ATPase (SERCA) protein are present in skeletal muscle and responsible for the active transport of Ca^2+^ from the sarcoplasm to the sarcoplasmic reticulum. SERCA1 is expressed exclusively in fast-twitch fibres, while SERCA2 is the variant encountered in cardiac muscle and slow-twitch fibres [64]. Research has shown increased muscle SERCA1 and SERCA2 content after sprint training [65] and SERCA1 after 23 days of unilateral lower limb suspension [66]. In contrast, reduced SERCA1 [67,68] and SERCA2 [69] have been reported after endurance training in humans. Although an association between reduced SERCAs protein expression and cycling efficiency has been reported [69], no relationship was observed in the current study between VO_2_/W during the MFR test and the expression of either SERCA or the combination of the two. Our results show that the aerobic component of the MFR associates positively with the expression of SERCA2 and negatively with the expression of SERCA1, implying that the aerobic component of the MFR is determined by the muscle fibre properties linked to aerobic metabolism and endurance. More specifically, our results suggest that an enhanced capacity to reuptake Ca^2+^ in type I fibres is a contributing factor to the aerobic component of the MFR. Moreover, since high oxidative stress may reduce the SERCAs' activity [70], a higher amount of SERCA2 may allow better resisting oxidative stress's detrimental effects on Ca^2+^ homeostasis in the slow-twitch fibres and explain a higher aerobic MFR as observed in the current experiments.

Another novelty of this study is the measurement of muscle myoglobin content in a large sample of volunteers. The variance in myoglobin expression explained 2.8% of the variance in the aerobic MFR, being its standardized estimate negative. This seems counterintuitive, given the role of myoglobin in intracellular O_2_ diffusion [71]. However, myoglobin overexpression may result in nitrosative stress due to the nitrite reductase activity of myoglobin and a low cellular PO_2_, as presumably reached during the occlusions in our experiments [72]. Excessive nitric oxide (NO) may inhibit mitochondrial respiration [73], limiting the aerobic component of the MFR.

Lastly, 2.7% of the variance in the aerobic component of the MFR was explained by Keap1 protein expression, such that the lower the expression of Keap1, the higher the aerobic component of the MFR. Likewise, Keap1 had a negative predictive value on the mean power developed during the MFR test. These findings concur with the enhanced endurance [11] and strength [74] observed in a genetic model of reduced expression of Keap1 in skeletal muscle. We surmise that lower availability of Keap1 in the rested state may facilitate greater levels of pSer^40^-Nrf2 and antioxidant enzymes [74] allowing a more efficient counteraction of RONS production during repeated cycles of ischaemia-reperfusion. This agrees with the observed greater increase in antioxidant proteins in slow than fast-twitch muscles in genetic models of Keap1 deletion [74].

### 4.3 The glycolytic component of the functional reserve is related to mechanisms regulating the muscle's antioxidant capacity

The O_2_ deficit incurred in the MFR can be predicted by the protein expression levels of pSer^40^-Nrf2, which explains 23% of the variance of the MFR. The phosphorylation of Nrf2 at Serine [40] facilitates its translocation to the nucleus and the subsequent gene transactivation [75,76]. Nrf2 can be linked to exercise performance by several mechanisms. First, rodents overexpressing Nrf2 have increased endurance [6,74] and nuclear Nrf2 protein content after prolonged running [7]. Although the molecular mechanisms by which Nrf2 may increase performance remain elusive, they are likely linked to an improved antioxidant capacity [74]. For example, Nrf2 increases the expression of the TP53-Induced Glycolysis and Apoptosis Regulator (TIGAR) [77], which promotes the production of nicotinamide adenine dinucleotide phosphate (NADPH) for glutathione (GSH) regeneration, resulting in enhanced antioxidant capacity in the resting muscle [78]. It has also been shown that Nrf2 elicits the expression of 6-phosphofructo-2-kinase/fructose-2,6-biphosphatase 3 (PFKFB3) [79], which catalyses the conversion of fructose-6-phosphate to fructose-2,6-bisphosphate -a potent allosteric activator of 6-phosphofructokinase-1 (PFK-1). Experiments with cancer cells also indicate that Nrf2 may upregulate the expression of HK2 [80], which may allow higher glycolytic rates. Moreover, increased HK2 has been shown to bind to mitochondria, where it secures a steady ADP formation, contributing to maintaining the membrane potential and reducing ROS production [81].

A higher SOD2 has been shown to protect against ischaemia-reperfusion [82]. In the present exercise model, the skeletal muscle is submitted to repeated episodes of ischaemia with full muscle deoxygenation followed by fast reperfusion upon resumption of exercise. We surmise that SOD2 helps preserve mitochondrial function and therefore enables the utilization of O_2_ at close-to-maximal rates even in states of apparently unsurmountable muscle fatigue. This interpretation is also supported by the linear association observed between VO_2_max per kg of LLM and SOD2. Moreover, we have recently shown that the oral administration of polyphenols with potent direct (by quenching RONS) and indirect (by inhibiting XO and NOX, two of the main sites of RONS production during high-intensity exercise [3]) antioxidant effects enhance O_2_ consumption and performance during repeated sprint exercise when subjects are requested to sprint maximally with normal blood flow after a short period of post-exhaustive exercise ischaemia [17,19].

RONS have been implicated in muscle fatigue [83], and RONS production is facilitated by the acidification caused by strong activation of the glycolysis in the presence of Fe^2+^ through the Fenton reaction [84]. Both catalase and myoglobin may counteract the deleterious effects of RONS produced during high-intensity exercise, particularly when accompanied by high glycolytic rates, as in the present exercise model. This concurs with the association between blood lactate concentration at the end of the MFR and basal catalase and myoglobin protein expressions reported here. Both proteins are crucial in redox regulation in skeletal muscle [[85], [86], [87], [88]].

Since part of the functional reserve could be explained by mechanisms delaying central fatigue including the agonistic capacity to overcome fatigue, we also examined whether differences in brain oxygenation could explain our findings.

### 4.4 Brain and vastus lateralis oxygenation role in the MFR

Brain oxygenation depends on arterial O_2_ content, cerebral blood flow and cerebral metabolic rate. In the present investigation, frontal lobe oxygenation explained 6.1% of the variability in the aerobic component of MFR. We hypothesized that subjects with higher brain oxygenation levels would perform better during repeated supramaximal exercise to exhaustion. Paradoxically, the results point in the opposite direction, as indicated by the negative coefficient (see the corresponding standardized estimate in Table 4). The latter implies that subjects with a higher MFR achieve greater levels of brain deoxygenation during intense exercise. Thus, we believe that the lower frontal lobe oxygenation observed in the subjects with a greater aerobic component of the functional reserve is a consequence and not the cause since humans can tolerate much lower levels of brain oxygenation during sprint exercise in severe acute hypoxia when assessed using the same equipment and laboratory conditions than in the present experiments [50].

However, the TOI *vastus lateralis* O_2_ extraction index was linearly associated with the MFR in kJ·kg LLM^−1^. The TOI *vastus lateralis* O_2_ extraction index depends on the balance between O_2_ delivery and O_2_ utilization, such that a higher muscle perfusion for a given muscle O_2_ demand with results in a greater the TOI *vastus lateralis* O_2_ extraction index during the MFR test, facilitating both O_2_ delivery and removal of metabolites to attenuate fatigue.

### 4.5 The functional reserve is similar in males and females matched for aerobic and anaerobic fitness

In the present investigation males and females had a similar VO_2_max normalized to LLM, indicating similar aerobic fitness. Likewise, no sex differences were observed in anaerobic power and capacity, as reflected by similar peak and mean power output per kg of LLM in the Wingate test, as well as MAOD values normalized to LLM and blood lactate concentrations after the MFR test. In our previous study we observed that males and females achieved similar values of functional reserve in kJ·kg LLM^−1^ after two bouts of repeated supramaximal exercise with total occlusion of the circulation during the recovery periods [16]. The present investigation confirms our previous findings and shows that when exercise bouts are repeated until the subjects cannot perform an additional bout, males and females have a similar functional reserve. This occurred even though females had a higher percentage of MHC I and higher basal expression of SERCA2 and CSQ2 in their *vastus lateralis* than males, while no significant sex-differences were observed in the rest of muscle proteins analysed in the present investigation.

### 4.6 Limitations

In the present investigation substrate-level phosphorylation has been assessed indirectly, assuming constant muscle efficiency during high-intensity exercise [54,89]. Although a decreased muscle efficiency with fatigue has been reported *in vitro* [90], the impact of fatigue on muscle efficiency in human skeletal muscle is not conclusive [91]. Moreover, at physiological muscle temperatures the effect of fatigue on muscle efficiency is likely small in humans [15,89,92]. Had muscle efficiency deteriorated with fatigue, we could have underestimated the actual contribution of substrate level phosphorylation to the functional reserve.

Twenty of our volunteers were able to perform six bouts of exercise, i.e., they showed a functional reserve after five occlusions. Thus, the actual functional reserve in these subjects could have been underestimated. Since the contribution of the last bout to the overall functional reserve was negligible in most subjects, and several did the last bout at 100% of VO_2_max, we surmise that this potential underestimation would be negligible. Nevertheless, it would be worthwhile to extend the number of repetitions in future experiments until all subjects cannot re-start pedalling at 100% of VO_2_max.

Finally, this study is the first to analyse the skeletal muscle responses to repeated episodes of ischaemia-reperfusion in humans. Therefore, only young healthy subjects were included, which were quite homogeneous regarding physical fitness. It remains unknown whether elite athletes have increased (or decreased) functional reserve and how ageing and diseases could affect the maximal functional reserve.

In summary, a large metabolic reserve exists in skeletal muscle at exhaustion which has an obligatory component relying on a reserve in glycolytic capacity and a putative component which depends on oxidative phosphorylation. We have shown that this functional reserve is similar in males and females matched for physical fitness. Moreover, we have identified several phenotypic muscle characteristics that explain the aerobic and glycolytic components of the functional reserve and found that the MFR is associated with muscle characteristics determining antioxidant signalling, calcium handling, and O_2_ transport and diffusion. Further manipulation of these phenotypic features, through, for example, exercise training/detraining or administering antioxidants, will be required to gain insight into the mechanisms determining the maximal functional reserve.

## Disclosure summary

The authors have nothing to disclose.

## Declaration of competing interest

The authors declare that they have no known competing financial interests or personal relationships that could have appeared to influence the work reported in this paper.

## Data Availability

The data that support the findings of this study are not openly available due to reasons of sensitivity and are available from the corresponding author upon reasonable request
