# Supplementary material for: Determinants of the maximal functional reserve during repeated supramaximal exercise by humans: The roles of Nrf2/Keap1, antioxidant proteins, muscle phenotype and oxygenation
Source: Redox Biol. 2023 Aug 22;66:102859. doi: 10.1016/j.redox.2023.102859 (PMC10491831; doi:10.1016/j.redox.2023.102859)
Supplement: Multimedia component 1 [file mmc1.docx]

**Supplementary Table 1: Detailed description of Western blotting antibodies and procedures.**

| **Antibody** |  | **Manufacturer company** |  | **Catalogue number** |  | **Molecular weight (kDa)** |  | **Gel %** |  | **Protein loaded**  **(μg)** |  | **Blotting transfer time (min)** |  | **Blocking reagent** |  | **Primary antibody concentration** |  | **Secondary**  **antibody concentration** |
| --- | --- | --- | --- | --- | --- | --- | --- | --- | --- | --- | --- | --- | --- | --- | --- | --- | --- | --- |
| Nrf2 |  | Abcam |  | ab62352 |  | 100 |  | 12.5 |  | 12.5 |  | 90 |  | BSA 4% |  | 1:1500 |  | 1:5000 |
| pSer40 Nrf2 |  | Abcam |  | ab76026 |  | 100 |  | 10 |  | 15 |  | 90 |  | BSA 4% |  | 1:5000 |  | 1:5000 |
| Keap1 |  | Proteintech |  | 10503-2-AP |  | 70 |  | 10 |  | 7.5 |  | 90 |  | BSA 4% |  | 1:3000 |  | 1:10000 |
| Catalase |  | Cell Signaling |  | 14097 |  | 60 |  | 10 |  | 12.5 |  | 90 |  | Blotto 5% |  | 1:2000 |  | 1:5000 |
| SOD1 |  | Abcam |  | ab16831 |  | 17 |  | 15 |  | 12.5 |  | 90 |  | BSA 4% |  | 1:2000 |  | 1:5000 |
| SOD2 |  | Cell Signaling |  | 13141 |  | 22 |  | 15 |  | 12.5 |  | 90 |  | BSA 4% |  | 1:20000 |  | 1:10000 |
| Calsequestrin1 |  | Sigma-Aldrich |  | C0618 |  | 55 |  | 10 |  | 1.5 |  | 90 |  | Blotto 5% |  | 1:30000 |  | 1:20000 |
| Calsequestrin2 |  | Sigma Aldrich |  | C3868 |  | 55 |  | 12.5 |  | 3 |  | 90 |  | BSA 4% |  | 1:5000 |  | 1:5000 |
| SERCA1 |  | Sigma-Aldrich |  | WH0000487M1 |  | 110 |  | 10 |  | 1.5 |  | 90 |  | Blotto 5% |  | 1:50000 |  | 1:20000 |
| SERCA2 |  | Sigma-Aldrich |  | S1439 |  | 110 |  | 10 |  | 3 |  | 90 |  | BSA 4% |  | 1:5000 |  | 1:5000 |
| Phosphofructokinase1 (PFKM) |  | Proteintech |  | 55028-1-AP |  | 85 |  | 10 |  | 1.5 |  | 90 |  | BSA 4% |  | 1:3000 |  | 1:20000 |
| NDUFB8 (CI) |  | Abcam |  | ab110411 |  | 18 |  | 4-20 |  | 8.5 |  | 90 |  | Blotto 5% |  | 1:2000 |  | 1:5000 |
| SDHB (CII) |  | Abcam |  | ab110411 |  | 29 |  | 4-20 |  | 8.5 |  | 90 |  | Blotto 5% |  | 1:2000 |  | 1:5000 |
| UQCRC2 (CIII) |  | Abcam |  | ab110411 |  | 38 |  | 4-20 |  | 8.5 |  | 90 |  | Blotto 5% |  | 1:2000 |  | 1:5000 |
| COXII (CIV) |  | Abcam |  | ab110411 |  | 22 |  | 4-20 |  | 8.5 |  | 90 |  | Blotto 5% |  | 1:2000 |  | 1:5000 |
| ATP5A (CV) |  | Abcam |  | ab110411 |  | 54 |  | 4-20 |  | 8.5 |  | 90 |  | Blotto 5% |  | 1:2000 |  | 1:5000 |
| Myoglobin |  | Cell Signaling |  | D2F5X |  | 17 |  | 15 |  | 0.3 |  | 60 |  | BSA 4% |  | 1:20000 |  | 1:50000 |
| Citrate Synthase |  | Proteintech |  | 16131-1-AP |  | 45 |  | 10 |  | 1.5 |  | 90 |  | BSA 4% |  | 1:3000 |  | 1:10000 |
| Total NFκB p65 |  | Cell Signaling |  | CS3034 |  | 65 |  | 10 |  | 15 |  | 90 |  | BSA 4% |  | 1:2000 |  | 1:10000 |
| pSer^536^ NFκB p65 |  | Cell Signaling |  | CS3033 |  | 65 |  | 10 |  | 12.5 |  | 90 |  | BSA 4% |  | 1:5000 |  | 1:5000 |
| Total p38 MAPK |  | Cell Signaling |  | CS9212 |  | 38 |  | 10 |  | 15 |  | 90 |  | Blotto 5% |  | 1:2000 |  | 1:10000 |
| pThr180/Tyr182 p38 MAPK |  | Cell Signaling |  | CS9211 |  | 38 |  | 10 |  | 10 |  | 90 |  | BSA 4% |  | 1:2000 |  | 1:10000 |
